# The functional divergence of homologous *GPAT9* genes contributes to the erucic acid content of *Brassica napus* seeds

**DOI:** 10.1186/s12870-024-04734-0

**Published:** 2024-01-24

**Authors:** Hongbo Liu, Jinbo Zhu, Bingxin Zhang, Qingyang Li, Cui Liu, Qian Huang, Peng Cui

**Affiliations:** 1 The Key Laboratory for Quality Improvement of Agricultural Products of Zhejiang Province, College of Advanced Agricultural Sciences, Zhejiang A & F University, Hangzhou, 311300 China; 2 Institute of Crop Science and Zhejiang Key Laboratory of Crop Germplasm, Zhejiang University, Hangzhou, 310058 China

**Keywords:** *Brassica napus*, Glycerol 3-phosphate acyltransferase 9, Yeast genetic complementation, Haplotype, Erucic acid

## Abstract

**Background:**

The early allopolyploid *Brassica napus* was a hybrid of two *Brassica* species, that had undergone a whole genome duplication event followed by genome restructuring, including deletions and small scale duplications. A large number of homologous genes appeared functional divergence during species domestication. Due to the high conservation of *de novo* glycerolipid biosynthesis, multiple homologues of glycerol-3-phosphate acyltransferases (GPATs) have been found in *B*. *napus*. Moreover, the functional variances among these homologous GPAT-encoding genes are unclear.

**Results:**

In this study, four *B*. *napus* homologous genes encoding glycerol-3-phosphate acyltransferase 9 (BnaGPAT9) were characterized. Although a bioinformatics analysis indicated high protein sequence similarity, the homologues demonstrated tissue-specific expression patterns and functional divergence. Yeast genetic complementation assays revealed that *BnaGPAT9-A1/C1* homologues but not *BnaGPAT9-A10/C9* homologues encoded functional GPAT enzymes. Furthermore, a single nucleotide polymorphism of *BnaGPAT9-C1* that occurred during the domestication process was associated with enzyme activity and contributed to the fatty acid composition. The seed-specific expression of *BnGPAT9*-*C1*^*1124A*^ increased the erucic acid content in the transformant seeds.

**Conclusions:**

This study revealed that *BnaGPAT9* gene homologues evolved into functionally divergent forms with important roles in erucic acid biosynthesis.

**Supplementary Information:**

The online version contains supplementary material available at 10.1186/s12870-024-04734-0.

## Introduction

Oilseed rape (*B. napus* L.) is an economically important crop that provides more than 13% of the world’s vegetable oil production [1]. *B. napus* is also a potential resource for the sustainable production of renewable bioenergy. However, to solve the challenges faced in the genetic improvement of seed fatty acid components, it is essential to fully understand oil biosynthesis [2, 3]. It is known that the GPATs as an initial and rate-limiting enzymes in the acylation reactions of *de novo* triacylglycerol (TAG) biosynthesis [2, 4, 5]. In previous study, the AtGPAT9 was identified to encode an endoplasmic reticulum *sn-1* GPAT in TAG synthesis of seed [6, 7]. In addition, the decrease of oil content and increase of total polyunsaturated fatty acids in the seeds of the *AtGPAT9* knockout mutant independent lines [8]. To date, only a few membrane-bound GPATs in *B. napus* (BnaGPATs) have been identified because of inconvenient purification and detection methods [9, 10]. 

The allotetraploid *B. napus* originated from the interspecific hybridization of *B. oleracea* and *B. rapa* [11, 12]. During evolution, early allopolyploidy and chromosome duplication resulted in genes with multiple homologues, which increases the difficulty of studying homologous gene functions. Moreover, functional divergence occurred in both paralogous and homoeologous genes during long-term domestication [9]. In addition, the redundancy of homologous genes affects transcription and functional divergence in genetic engineering with polyploid backgrounds. Fortunately, *B*. *napus* genomes and pan-genome have been assembled as whole-genome sequencing research has increased, and provide large amounts of data that can be used to mine for genes with vital quality and agronomic traits [13–16]. However, it should be noted that caution in applying the sequence data from progenitor genomes rearrangements (*B*. *rapa* and *B*. *oleracea*), even mostly functionally conserved in homologous gene [17]. Fatty acid elongase (*FAE1* gene-encoded) catalyses two successive condensation reactions using oleoyl-CoA as the substrate in erucic acid biosynthesis [18]. Loss of function in *FAE* genes in domesticated *B. napus* results in rapeseed varieties with low erucic acid levels [19], whereas the contributions of GPATs to the *de novo* biosynthesis of fatty acids are unknown. Furthermore, it also unknown if there exist GPATs lacking the conserved acyltransferase domains in plants. Therefore, we have constructed a yeast genetic complementation system for the screening of eukaryotic GPATs [20]. By constructing the exogenous gene into the yeast expression vector yADH1-pYES2-KanV2 and transforming it into the yeast strain ZAFU1, we can quickly identify whether the exogenous gene has acyltransferase activity based on whether it can restore the growth of yeast on a medium supplemented with glucose. In this study, to examine the homologous functional divergence in glycerolipid biosynthesis, four homologous *BnaGPAT9*-encoded genes were characterized by yeast genetic complementation and transformation of seed-specific expression.

## Results

### Characterization of homologous *GPAT9* genes in *B. Napus*

Four homologous *GPAT9* genes (i.e., *BnaGPAT9*-*A1*, *C1*, *A10*, *C9*) was amplified from *B*. *napus* (advanced line 21L10). All of these sequencing data have been uploaded into the NCBI repository, and the GenBank accession numbers of *BnaGPAT9*-*A1* (OR521147), *C1* (OR521149), *A10* (OR521148), *C9* (OR536417) are listed in Supplementary Table S1. Table 1 shows the CDS and protein sequence identity of the four *BnaGPAT9* genes, and 97.2% similarity was found between the orthologous gene pairs *BnaGPAT9*-*A1* and *BnaGPAT9*-*C1* and *BnaGPAT9*-*A10* and *BnaGPAT9*-*C9*. Moreover, protein sequence similarities of 100% and 98.4% were found between *A1* and *C1* and between *A10* and *C9*, respectively. The sequence differences in CDS and proteins among the four homologues are illustrated in Supplementary Figure S1 and S2, respectively.

**Table 1:** CDS and protein sequence identity of the four homologous *BnaGPAT9* genes

	BnaGPAT9-A10		BnaGPAT9-C1		BnaGPAT9-C9	
	CDS	Protein	CDS	Protein	CDS	Protein
BnaGPAT9-A1	87.6%	92.8%	97.2%	100%	88.0%	92.8%
BnaGPAT9-A10	-	-	87.7%	92.8%	97.2%	98.4%
BnaGPAT9-C1	-	-	-	-	88.2%	92.8%

### Identification of BnaGPAT9 activity

**Fig. 1:**
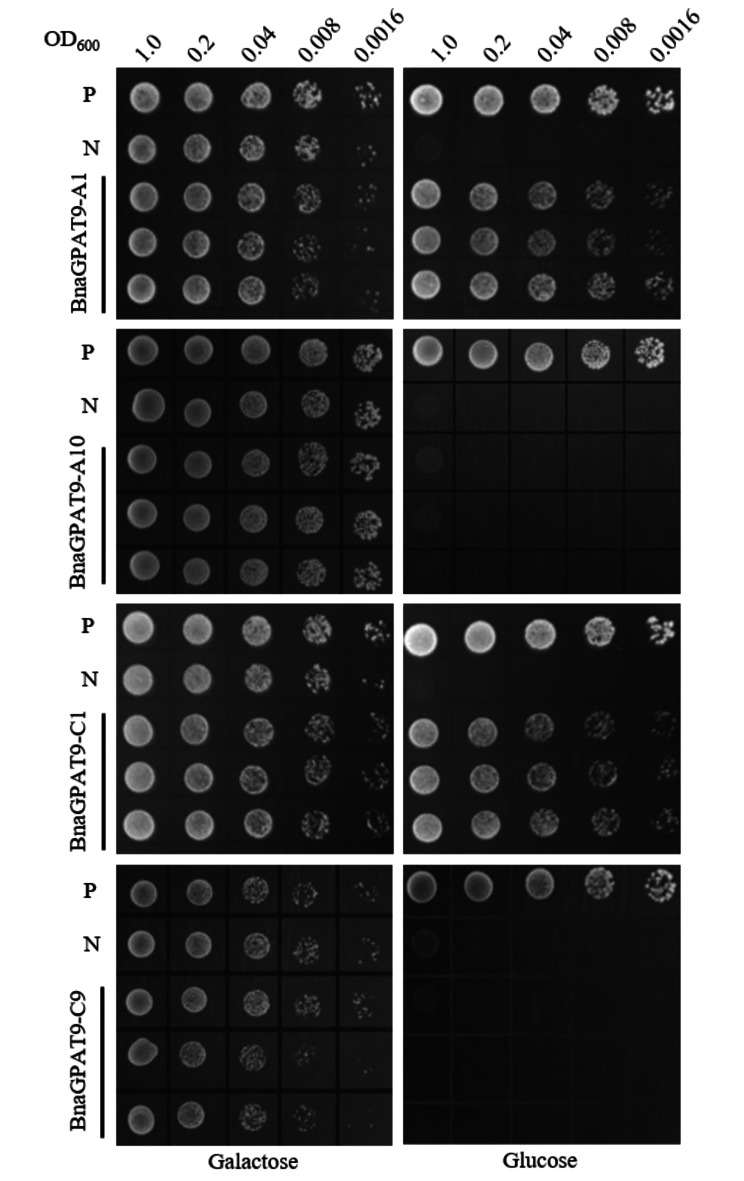
Only one pair of homeologous *BnaGPAT9* genes rescues yeast growth under glucose. Yeast genetic complementation with heterologous expression of *BnaGPAT9*-*A1*/*C1* could rescue the mutant strain ZAFU1 growth on SC-Ura-His-Leu + glucose solid medium, but *BnaGPAT9*-*A10*/*C9* did not rescue growth. P: positive control (*glycerol-3-phosphate acyltransferases 1* gene of *Saccharomyces cerevisiae*, *ScGAT1*); N: negative control (empty vector). The independent colonies were serially diluted (1:5) to an initial optical density (OD_600_) of 1.0

The full-length CDS of *BnaGPAT9*-*A1*/*C1*/*A10*/*C9* were cloned into the vector yADH1-pYES2-Kan V2 (Supplementary Fig. S3), and were transformed into the yeast mutant strain (ZAFU1) to identify acyltransferase activity. The exogenous gene (*AtGPAT1*) in episomal vector was driven by galactose induced promoter, that can support the growth of the ZAFU1 on SC-Ura-His-Leu + galactose medium. On the other hand, the candidate *GPAT* in another episomal vector was driven by glucose induced promoter, that can rescue the growth of the ZAFU1 on SC-Ura-His-Leu + glucose medium. According to yeast genetic complementation, BnaGPAT9-A1 and BnaGPAT9-C1 rescued the growth of ZAFU1 on SC-Ura-His-Leu + glucose solid medium, but BnaGPAT9-A10 and BnaGPAT9-C9 did not rescue growth (Fig. 1). Thus, *BnaGPAT9-A1*/*C1* have acyltransferase activity.

### Tissue-specific expression of *BnaGPAT9*-*A1*/*C1*

**Fig. 2:**
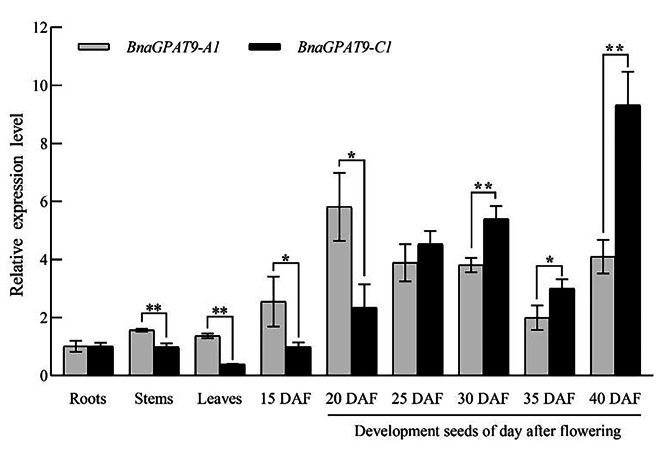
Relative expression patterns of *BnaGPAT9-A1/C1* in different tissues of ‘Zhongshuang 11’. The relative expression level of *BnaGPAT9-A1*/*C1* genes were higher in development seeds than that of in roots, stems, and leaves. At early stages of development seeds, the *BnaGPAT9-A1* relative expression level was higher than that of *BnaGPAT9-C1*, whereas on the contrary at later stages of development seeds. The values are the means ± SDs, *n* = 3

The expression patterns of *BnaGPAT9-A1*/*C1* were analysed by qRT‒PCR in roots, stems, leaves, and developing seeds (Supplementary Fig. S4). The relative expression of *BnaGPAT9-A1* in seeds at early stages of development (15 and 20 days after flowering) was higher than that of *BnaGPAT9-C1*, whereas at later stages of development (25, 30, 35, and 40 days after flowering), the relative expression of *BnaGPAT9-C1* was higher than that of *BnaGPAT9-A1* (Fig. 2). Analyses of the expression patterns shown that these two genes transcript was associated with seed development.

### Effect of single nucleotide polymorphisms on *BnaGPAT9-C1* enzyme activity

**Fig. 3:**
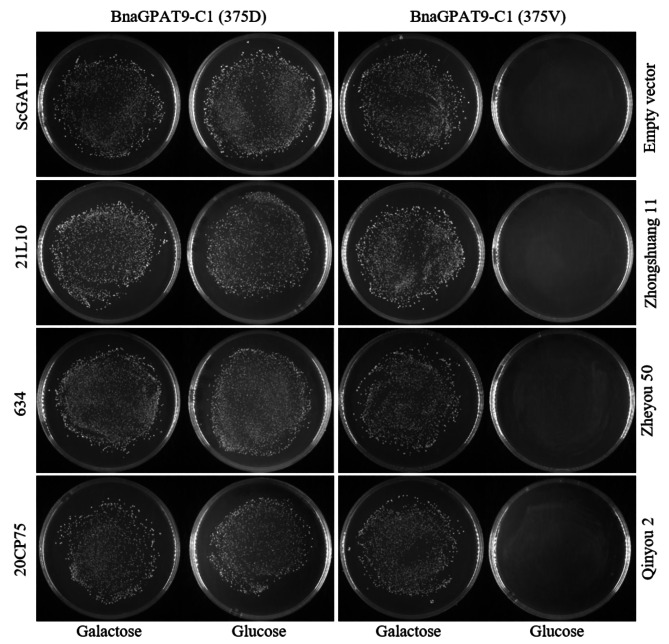
The advanced lines 21L10, 20CP75 and 634 with BnaGPAT9-C1 (GA^1124^C encodes aspartic acid 375D) have acyltransferase activity that can rescues the ZAFU1 growth on the SC-Ura-His-Leu + glucose solid medium, whereas commercial varieties Qinyou2, Zheyou50 and Zhongshuang11 with BnaGPAT9-C1 (GT^1124^C encodes valine 375 A) did not. ScGAT1: *S. cerevisiae* glycerol-3-phosphate acyltransferase 1; Empty vector: yADH1-pYES2-Kan V2; 21L10, 634, 20CP75: advanced lines; Zhongshuang 11, Zheyou 50, Qinyou 2: commercial varieties

**Fig. 4:**
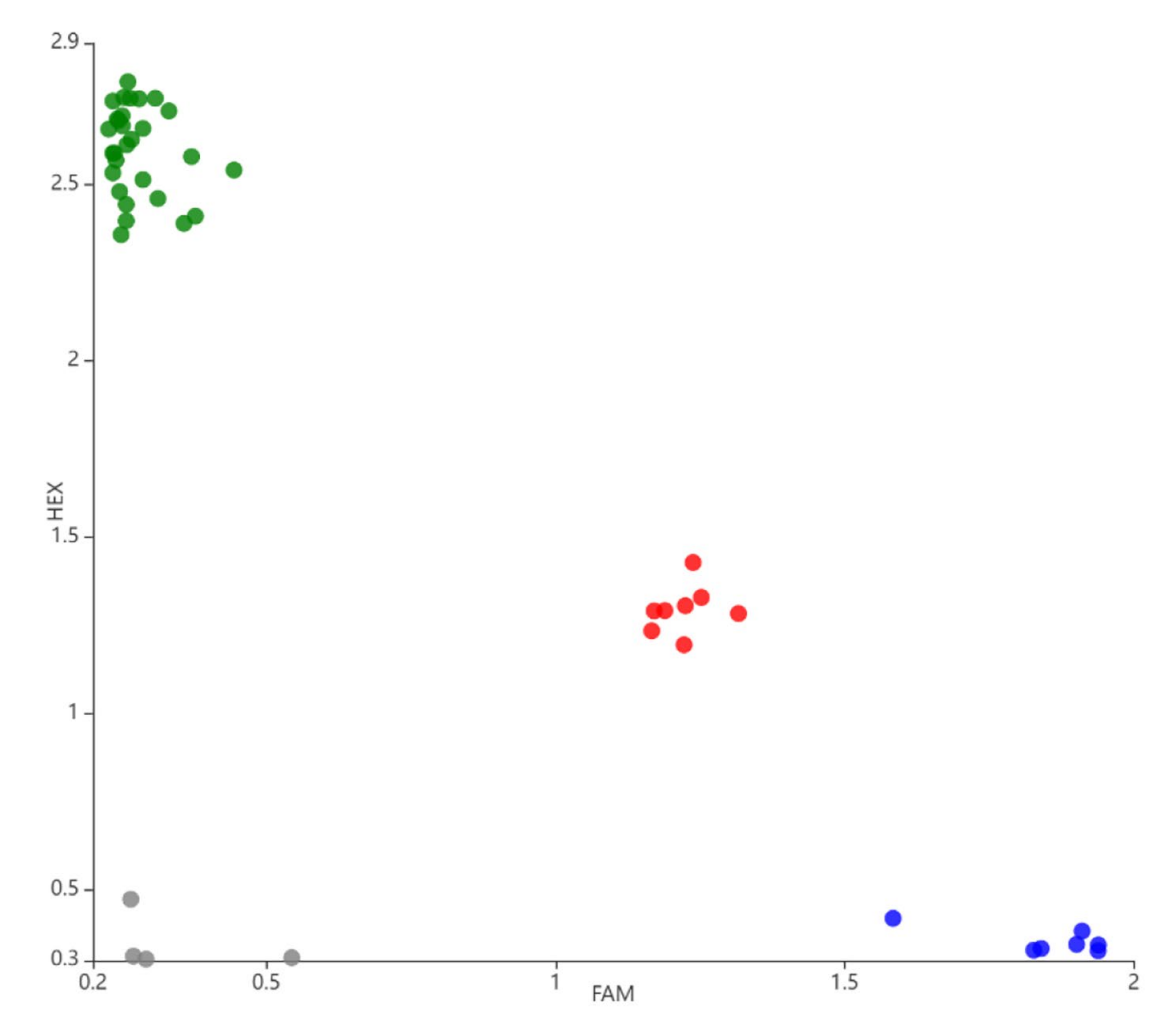
44 genotypes of *B. napus* were divided into three haplotypes with single nucleotide polymorphism in position of the *BnaGPAT9-C1*^*1124*^ by a penta-primer amplification refractory mutation system (PARMS). Blue dots (seven materials) represent homozygous haplotypes (A/A); red dots (eight materials) represent heterozygous haplotypes (A/T); green dots (twenty nine materials) represent homozygous haplotypes (T/T); grey dots represent negative controls. HEX: green fluorescence universal primer; FAM: blue fluorescence universal primer

A single nucleotide polymorphism (SNP) occurred in *BnaGPAT9-C1* (GA^1124^C or GT^1124^C) that induced a change from aspartic acid (D) into valine (V) at the 375 aa site. The sequencing data of *BnaGPAT9-C1* from commercial varieties Qinyou2 (OR536420), Zheyou50 (OR536421), Zhongshuang11 (OR536422) and advanced lines 20CP75 (OR536418), 634 (OR536419) has been uploaded into the NCBI repository (Supplementary Table S1). Notably, the amino acid sequence with 375 V exhibited no GPAT enzyme activity to rescue the growth of ZAFU1 on SC-Ura-His-Leu + glucose solid medium (Fig. 3). Furthermore, based on the SNP, 44 materials (14 commercial varieties and 30 advanced lines) were classified into three haplotypes by the PARMS genotyping platform (Fig. 4). The results shown that 18.2% and 65.9% of the varieties/lines belonged to the H2 (A/T heterozygous) and H3 (T/T homozygous) haplotypes, respectively (Table 2).

**Table 2:** 44 commercial varieties/advanced lines were divided into three haplotypes by a single nucleotide genotyping at 1124 bp site of the *BnaGPAT9-C1* gene in *B*. *napus*. Haplotype analysis of *BnaGPAT9-C1*^*1124*^ using a penta-primer amplification refractory mutation system (PARMS)

Haplotype	Genotype	Variety (Line)	Proportion
H1	A/A	0 (7)	15.9%
H2	A/T	5 (3)	18.2%
H3	T/T	9 (20)	65.9%

### Association of haplotype and erucic acid (C22:1) content in seeds

**Fig. 5:**
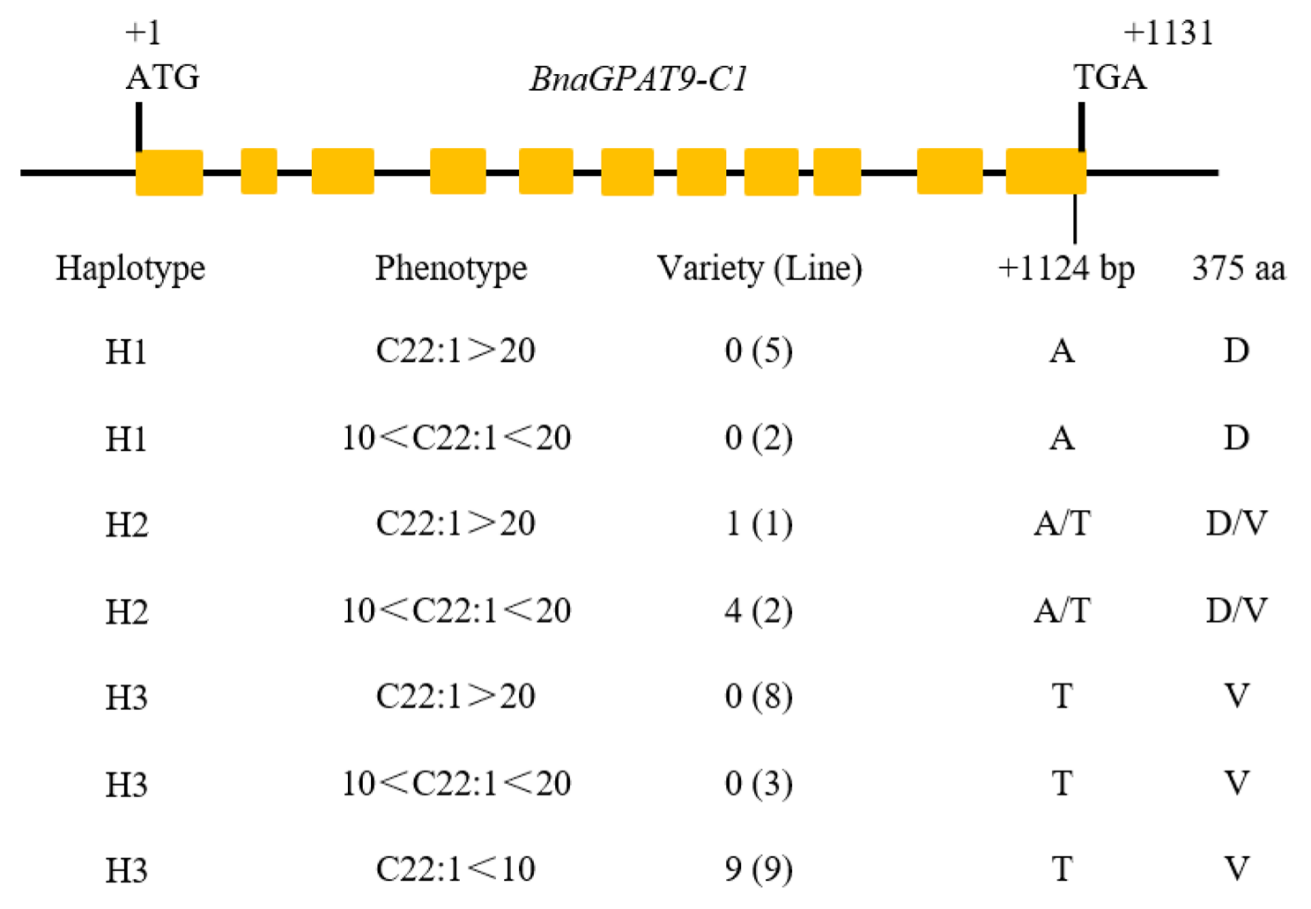
Distribution of three haplotypes with different erucic acid content. In H1 haplotype, the erucic acid content of all advanced lines was more than 10%. In H3 haplotype, the erucic acid content of all commercial varieties was less than 10%. H1: homozygous haplotype (A/A), H2: heterozygous haplotype (A/T), H3: homozygous haplotype (T/T)

The seed fatty acid compositions were analysed by gas chromatography in 44 genotypes of *B. napus* (Supplementary Table S2). The analysis showed that the erucic acid content of all H1 haplotypes (A/A) and H2 haplotypes (A/T) was higher than 10%, whereas that of 62.1% (18/29) of the H3 (T/T) haplotypes was less than 10%, especially in all commercial varieties (Fig. 5 and Supplementary Fig. S5). In addition, 11 advanced lines of the H3 haplotype had erucic acid contents higher than 10% (Fig. 5). These results implied that GPAT enzyme activity could increase the erucic acid (C22:1) content of seeds and other regulatory pathways in erucic acid biosynthesis for genetic improvement.

### Seed-specific expression of *BnaGPAT9-C1*^*1124A*^ for regulating the erucic acid content in ‘Zhongshuang 11’ seeds

**Fig. 6:**
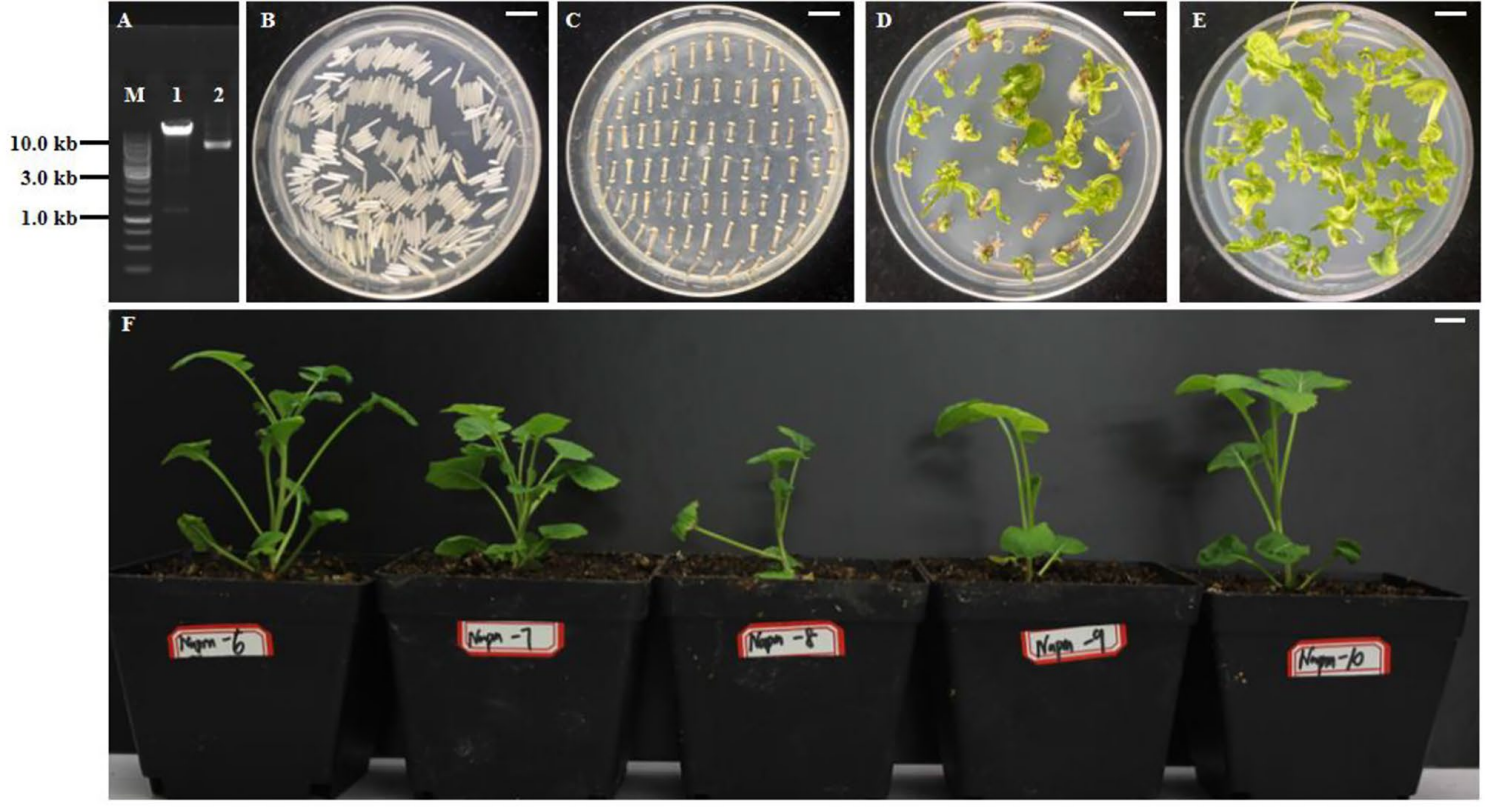
Construction of p*Napin*-*BnaGPAT9-C1*^*1124A*^-*Nos* transformants in the ‘Zhongshuang 11’ genetic background. (A) Identification of the recombinant vector. 1: Double enzyme digestion of the recombinant vector, the *BnaGPAT9-C1* fragment was 1131 bp; 2: no enzyme digestion of the recombinant vector; M: 1-kb DNA ladder. (B–F) Genetic transformation of hypocotyls. B: hypocotyl; C: callus; D and E: induced shoots; F: transgenic plantlets. Scale bar = 1 cm

**Fig. 7:**
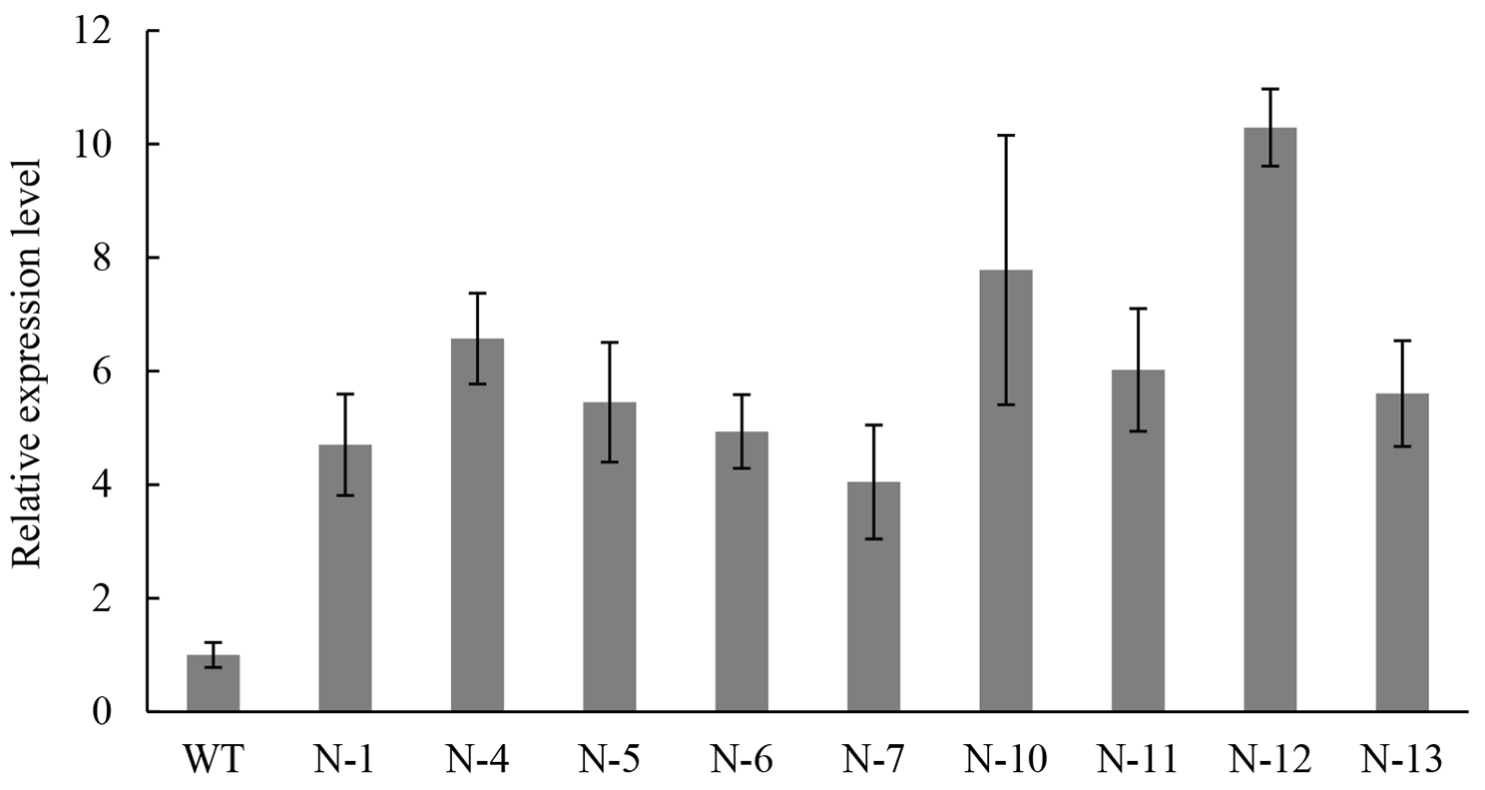
Relative expression level of p*Napin*-*BnaGPAT9-C1*^*1124A*^-*Nos* in independent T_0_ transgenic lines. The values are the means ± SDs, *n* = 3. WT: untransformed ‘Zhongshuang 11’ with the *BnaGPAT9-C1* homozygous haplotype (T/T) at the 1124 bp site. N-1 to N-13: independent p*Napin*-*BnaGPAT9-C1*^*1124A*^-*Nos* transformant lines in the ‘Zhongshuang 11’ genetic background

A 1131-bp target fragment was detected by double enzyme digestion in the recombinant vector p*Napin*-*BnaGPAT9-C1*^*1124A*^-*Nos* (Fig. 6A) which were used for *A. tumefaciens*-mediated genetic transformation. The morphology of explants, calli, induced shoots and transgenic plantlets at various stages of regeneration is shown in Fig. 6B and F. In seed-specific expression of *BnaGPAT9*-*C1*^*1124A*^ transformations, 13 positive transformants were obtained by specific PCR identification, and a 1027-bp target fragment amplified (Supplementary Fig. S6). Moreover, the expression level of *BnaGPAT9*-*C1*^*1124A*^ in the transformants was higher than that in the wild type, as revealed by qRT‒PCR (Fig. 7). The fatty acid content of T_1_ seeds of four transformants (lines 6, 11, 12, and 13) was analysed, and all of the components are shown in Table 3. The contents of oleic acid (C18:1) in the seeds of these four transgenic lines were significantly lower than that of the wild type, but the opposite results were found for the linolenic acid (C18:3) and erucic acid (C22:1) contents (Table 3).

**Table 3:** Analysis of the seed fatty acid composition of independent T_1_ transgenic lines with p*Napin*-*BnaGPAT9-C1*^*1124A*^-*Nos*

Line	Palmitic acid (C16:0)	Stearic acid (C18:0)	Oleic acid (C18:1)	Linoleic acid (C18:2)	Linolenic acid (C18:3)	Arachidonic acid (C20:0)	Eicosenoic acid (C20:1)	Erucic acid (C22:1)
WT	4.10b ± 0.05	2.25a ± 0.22	65.14a ± 0.91	18.81c ± 0.13	8.13b ± 1.05	0.58a ± 0.04	0.97a ± 0.03	0.02c ± 0.00
N-6	3.94b ± 0.16	2.24a ± 0.03	60.01b ± 0.55	20.83ab ± 0.39	10.20a ± 0.12	0.80a ± 0.30	1.09a ± 0.12	0.89a ± 0.00
N-11	6.86a ± 0.64	2.23a ± 0.75	53.88c ± 1.64	25.21a ± 2.93	10.11a ± 0.23	0.50a ± 0.05	0.99a ± 0.09	0.23b ± 0.01
N-12	6.47a ± 0.28	2.19a ± 0.37	54.19c ± 0.21	24.63a ± 0.05	10.49a ± 0.24	0.50a ± 0.01	1.22a ± 0.02	0.30b ± 0.08
N-13	4.56b ± 0.19	2.88a ± 0.04	61.97b ± 0.40	19.19c ± 0.57	9.54a ± 0.12	0.64a ± 0.04	1.03a ± 0.09	0.19b ± 0.01

The values are the means ± SDs, *n* = 3. The same lowercase letters within a column indicate no significant differences at *P* < 0.05. WT: untransformed ‘Zhongshuang 11’ with the *BnaGPAT9-C1* homozygous haplotype (T/T) at the 1124 bp site. N-6, N-11, N-12 and N-13: independent p*Napin*-*BnaGPAT9-C1*^*1124A*^-*Nos* transformant lines in the ‘Zhongshuang 11’ genetic background

## Discussion

With the sequencing of genomes that have undergone complex genomic changes such as whole/partial genome duplications, and interspecific hybridization we can study the functional evolution of duplicated genes. As a result, *B*. *napus* has become an important model for investigations of the consequences of polyploidy, which have provided intriguing insights into genome restructuring resulting from duplicated selective forces during crop evolution. However, little is known about the functional divergence between paralogous or homologous genes in the evolution of genomic segmental duplications. In this study, four homologous *BnaGPAT9* genes were identified. Based on sequence identity, we could able to determine the *BnaGPAT9-A1*/*A10* and *BnaGPAT9-C1*/*C9* origin from *B*. *rapa* and *B*. *oleracea*, respectively. Nevertheless, BnaGPAT9-A1/C1 exhibited GPAT enzyme activity and thus rescued the growth of the double knockout mutant strain (ZAFU1) in yeast genetic complementation assays, whereas BnaGPAT9-A10/C9 did not rescue ZAFU1 growth (Fig. 1). The results indicated that functional divergence occurred, and through this process, duplicated genes lose a redundant function during crop evolution [9, 21].

In addition, homologous genes have different developmentally regulated mechanisms in polyploidy crops [9, 22]. For example, the *BnaGPAT4-A1* and *BnaGPAT4*-*C1* shown significantly opposite expression level in maturing embryo and developing seed coat [9]. In our study, the seed-specific expression patterns indicated that *BnaGPAT9-C1* might be important in fatty acid biosynthesis during later stages of seed development (25–40 days after flowering), whereas *BnaGPAT9-A1* might be important in early stages (15–20 days after flowering) (Fig. 2). The possible mechanisms are supposed to be important for polyploid crops adaptation during the domestication processes [23].

In this study, an SNP of *BnaGPAT9-C1*^*1124T*^ resulted in GPAT activity that was too low to rescue ZAFU1 (Fig. 3) and produced a phenotype consisting of a low erucic acid content in seeds, especially in commercial varieties (Fig. 5). It is speculated that the emergence of multiple haplotypes may be due to the improved adaptability of crops during domestication processes [23]. Fatty acid analysis showed that transformants with seed-specific expression of *BnaGPAT9*^*1124A*^ exhibited an increase in the erucic acid content compared with that of ‘Zhongshuang 11’, a commercial variety with a low erucic acid content. In summary, the results of this study imply that *BnaGPAT9-A1/C1* has substrate preferences in triacylglycerol biosynthesis. For further step, we intend to use another line with high erucic acid as the genetic background or in vitro enzyme assays to check the preference of BnaGPAT9.

## Conclusions

Functional divergence in *BnaGPAT9* genes was identified using the yeast mutant strain ZAFU1 and expression pattern analysis. The *BnaGPAT9-A1/C1* homologues but not the *BnaGPAT9-A10/C9* homologues encoded functional GPAT enzymes. In addition, an SNP of *BnaGPAT9-C1* (A or T at a 1124-bp site) that occurred during evolution and domestication processes was associated with enzyme activity and contributed to the erucic acid content. Moreover, seed-specific expression of *BnaGPAT9*-*C1*^*1124A*^ increased the erucic acid content in the seeds of the transformants. Thus, this work will be of great interest to breeders working on the genetic and breeding improvement of oil crops.

## Materials and methods

### Plant materials

*B. napus* seeds of 44 genotypes (14 commercial varieties and 30 advanced lines) were used in the experiments. Twenty-five of the advanced lines were kindly provided by Prof. Weijun Zhou (Zhejiang University, Hangzhou, China), whereas four were provided by Dr. Qi Peng (Jiangsu Academy of Agricultural Sciences). Information on these materials is listed in Supplementary Table S3.

### Cloning and homology analysis of *GPAT9* genes from *B. Napus*

Fourteen-day-old seedlings of the *B. napus* advanced line ‘21L10’ were used to clone *BnaGPAT9-A1/C1/A10/C9* derived from the A and C genomes. The coding sequences of *GPAT9s* were amplified using the High-Fidelity Enzyme PrimeSTAR Kit (Takara, Japan) with the specific primers listed in Supplementary Table S4. The complementary DNA (cDNA) and protein sequence identity of the *GPAT9* genes were analysed using DNAMAN software version 9.0 (Lynnon BioSoft, San Ramon, CA, USA).

### Vector construction and yeast genetic complementation

To obtain heterologous expression, *BnaGPAT9* genes were cloned and inserted into yADH1-pYES2-Kan V2 vectors with glucose induction using the primers listed in Supplemental Table S4. In the yeast genetic complementation assay, the yeast mutant ZAFU1 (*BY4742*, *gat1Δgat2Δ*+[*pGAL1::AtGPAT1 LEU2*]) was used for screening acyltransferase activity. Subsequently, yeast genetic complementation procedures were conducted according to Lei et al. [20] and Liu et al. [24]. In a serial dilution (1:5) assay, a single clone was inoculated into SC-Ura-His-Leu + galactose or glucose medium with an initial optical density (OD_600_) of 1.0 and cultured for 5 d at 30 °C.

### Reverse-transcription quantitative PCR

Total RNA of ‘Zhongshuang 11’ was extracted using a TransZol Up Plus RNA Kit (Transgene, Shenzhen, China) and detected using a NanoDrop™ One (Thermo Fisher, Waltham, MA, USA). cDNA was synthesized using Hifair® 1st Strand cDNA Synthesis SuperMix for qPCR (Yeasen, Shanghai, China). The expression patterns of *BnaGPAT9-A1/C1* in roots, stems, leaves, and developing seeds (15, 20, 25, 30, 35, and 40 days after flowering) were determined by CFX Connect™ Optics Module (Bio-Rad, Hercules, CA, USA) with three biological replications. The 20-µl qRT‒PCR contained 10 µl of 2 x Hieff qPCR SYBR Green Master Mix (Yeasen, Shanghai, China), 0.004 nM forward primer, 0.004 nM reverse primer, and 9.2 µl of cDNA. The reaction program was initiated by predenaturation at 95°C for 5 min, and this step was followed by 40 cycles of denaturation (95°C for 10 s) and annealing (55°C for 30 s). The reference gene was *ubiquitin-conjugating enzyme 9* (accession no. XM_013800933) [9], which was used to normalize the expression levels of *BnaGPAT9-A1/C1*. To distinguish *BnGPAT9-A1*/*C1* by qPCR, primers were designed for the 3’ UTR and 5’ UTR differential regions of the *BnaGPAT9-A1* and *BnaGPAT9-C1* mRNA sequences, respectively. All primers are listed in Supplementary Table S4.

### Haplotype identification

Based on the SNP of *BnaGPAT9-A1/C1* in domestication, a penta-primer amplification refractory mutation system (PARMS) was designed to identify the haplotype in the commercial varieties and advanced lines. The standard operation procedure was performed according to Lu et al. [25], and the PARMS primers are listed in Supplementary Table S4.

### Seed fatty acid component analysis

To determine seed fatty acid components, 30.0 mg of seed samples were analysed using an Agilent 7890B gas chromatograph (Agilent, Santa Clara, CA, USA). The analysis was conducted according to Ichihara and Fukubayashi [26] with minor modifications. Samples were obtained from three biological duplicates of commercial varieties and advanced lines.

### Construction of *BnaGPAT9-C1*^*1124A*^ seed-specific expression transformants in Zhongshuang 11

*BnaGPAT9-C1*^*1124A*^ was cloned and inserted into a p1300-*Napin*-*Nos* vector with *Sac*I and *Pst*I. Genetic transformation with *Agrobacterium tumefaciens* GV3101 was conducted according to Liu et al. [27] with minor modifications. The T_0_ generation lines were confirmed by PCR, and T_1_ seeds from the *BnaGPAT9-C1*^*1124A*^ high-expression lines were harvested for fatty acid component analysis. All primers are listed in Supplementary Table S4.

### Statistical analyses

The seed fatty acid composition was determined on the basis of peak areas. The variance of the mean was analysed by least significant difference tests at the significance level of *P* ≤ 0.05. The relative expression level was calculated according to the 2^−ΔΔCt^ analysis method [28].

### Electronic supplementary material

Below is the link to the electronic supplementary material.

Supplementary Material 1

## Data Availability

The data that support this study are available in the article and accompanying online supplementary material.
