# Supplementary material for: The functional divergence of homologous GPAT9 genes contributes to the erucic acid content of Brassica napus seeds
Source: BMC Plant Biol. 2024 Jan 24;24:69. doi: 10.1186/s12870-024-04734-0 (PMC10807112; doi:10.1186/s12870-024-04734-0)
Supplement: Supplementary file 1 — Supplementary Material 1 [file 12870_2024_4734_MOESM1_ESM.docx]

**Supplementary Table S1** NCBI GenBank accession numbers of cloned *GPAT9-A1-*, *A10-*, *C1-*, and *C9*-encoding gene sequences from commercial varieties and advanced lines

| Gene | NCBI GenBank Accession Number |
| --- | --- |
| SUB13814607 BnaGPAT9-A1-CDS | OR521147 |
| SUB13814607 BnaGPAT9-A10-CDS | OR521148 |
| SUB13814607 BnaGPAT9-C1-CDS | OR521149 |
| SUB13828414 BnaGPAT9-C9-CDS | OR536417 |
| SUB13828414 20CP75-BnaGPAT9-C1-CDS | OR536418 |
| SUB13828414 634-BnaGPAT9-C1-CDS | OR536419 |
| SUB13828414 Qinyou2-BnaGPAT9-C1-CDS | OR536420 |
| SUB13828414 Zheyou50-BnaGPAT9-C1-CDS | OR536421 |
| SUB13828414 Zhongshuang11-BnaGPAT9-C1-CDS | OR536422 |

**Supplementary Table S2** Seed fatty acid composition of 44 commercial varieties/advanced lines

| Line No. | Palmitic acid  (C16:0) | Stearic acid  (C18:0) | Oleic acid  (C18:1) | Linoleic acid  (C18:2) | Linolenic acid  (C18:3) | Arachidonic acid  (C20:0) | Eicosenoic acid  (C20:1) | Erucic acid  (C22:1) |
| --- | --- | --- | --- | --- | --- | --- | --- | --- |
| 634 | 4.16±0.69 | 2.78±0.18 | 49.42±1.19 | 12.86±0.26 | 5.87±0.41 | 0.85±0.11 | 11.9±0.59 | 12.16±1.49 |
| 941 | 4.54±1.27 | 2.44±0.80 | 36.42±1.19 | 14.36±0.30 | 8.34±0.15 | 0.56±0.03 | 14.72±0.47 | 18.63±1.44 |
| 20CP75 | 4.84±1.17 | 2.16±0.91 | 11.98±0.89 | 15.44±0.47 | 8.77±0.27 | 0.69±0.06 | 3.83±1.35 | 52.29±1.58 |
| 20CP86 | 6.14±2.33 | 3.72±1.58 | 13.4±0.92 | 13.26±0.82 | 9.13±0.84 | 0.92±0.14 | 7.09±0.89 | 46.32±3.71 |
| 20CP102 | 5.45±2.98 | 3.43±1.93 | 30.82±5.14 | 13.34±0.59 | 8.18±0.85 | 0.82±0.09 | 13.22±2.90 | 24.74±8.64 |
| 20CP103 | 5.71±1.92 | 3.48±1.94 | 24.09±3.10 | 14.86±0.37 | 7.94±0.48 | 1.06±0.50 | 11.56±1.76 | 31.30±5.28 |
| 21L10 | 7.50±4.72 | 4.57±3.19 | 16.27±1.89 | 13.74±1.08 | 9.65±1.20 | 0.91±0.15 | 7.63±1.27 | 39.72±3.00 |
| 4095-2 | 8.25±1.21 | 6.03±0.34 | 33.32±1.40 | 9.66±0.87 | 6.27±0.43 | 0.97±0.10 | 15.77±0.52 | 19.72±1.71 |
| 4099 | 5.32±1.68 | 3.48±1.36 | 30.9±1.12 | 11.22±0.17 | 6.02±0.26 | 0.94±0.11 | 15.06±1.12 | 27.06±2.96 |
| 612 | 4.18±0.60 | 1.64±0.16 | 43.08±0.78 | 14.39±0.19 | 8.65±0.11 | 0.53±0.02 | 13.97±1.68 | 13.56±1.20 |
| Zhongyou 821 | 4.33±0.46 | 2.21±0.33 | 45.56±13.68 | 15.42±1.24 | 8.84±0.81 | 0.68±0.07 | 12.58±6.08 | 10.38±2.92 |
| Zhongyou 828 | 4.27±0.79 | 3.00±0.52 | 37.12±5.01 | 13.61±0.94 | 8.48±0.15 | 0.93±0.01 | 16.58±1.74 | 16.01±5.75 |
| Fengyou 737 | 5.47±1.61 | 3.81±0.89 | 37.57±3.53 | 12.05±0.84 | 7.20±0.28 | 0.93±0.09 | 14.97±1.75 | 17.99±1.97 |
| Zhengyou 1 | 3.89±0.45 | 2.50±0.41 | 33.31±6.63 | 12.36±0.37 | 8.64±0.30 | 0.73±0.05 | 18.02±2.09 | 20.56±9.93 |
| Zhengyouza 1 | 4.74±1.01 | 2.45±0.13 | 49.62±9.46 | 14.80±1.83 | 8.33±0.03 | 0.54±0.02 | 7.20±5.25 | 12.31±2.57 |
| 4017 | 4.45±0.96 | 2.26±0.35 | 30.07±1.15 | 12.05±0.17 | 7.49±0.59 | 0.69±0.03 | 14.74±0.33 | 28.26±2.48 |
| 4023 | 4.06±0.23 | 2.61±0.52 | 36.16±1.74 | 14.73±0.30 | 6.55±0.27 | 0.63±0.07 | 14.79±1.18 | 20.46±2.64 |
| 4054 | 4.28±0.35 | 2.20±0.10 | 31.94±4.01 | 14.82±0.31 | 8.43±0.19 | 0.71±0.05 | 15.86±0.16 | 21.76±4.07 |
| 4064 | 4.39±1.21 | 2.37±0.66 | 27.13±3.86 | 13.73±0.74 | 7.42±0.86 | 0.90±0.10 | 17.77±0.49 | 26.30±5.03 |
| 4066 | 3.63±0.11 | 2.28±0.19 | 45.51±7.46 | 13.55±1.20 | 7.06±0.21 | 0.74±0.09 | 14.16±4.19 | 13.07±5.05 |
| 4067 | 4.27±0.21 | 2.39±0.07 | 45.86±3.76 | 13.98±0.48 | 7.14±0.24 | 0.57±0.02 | 12.70±1.42 | 13.08±1.78 |
| 4068 | 4.67±0.69 | 3.39±0.66 | 56.71±4.56 | 15.05±0.45 | 7.70±0.44 | 0.72±0.05 | 6.52±0.55 | 5.23±2.40 |
| 4088 | 3.38±0.09 | 1.78±0.11 | 27.29±0.79 | 11.33±0.20 | 9.13±0.12 | 0.81±0.02 | 15.96±0.64 | 30.32±1.48 |
| 4089 | 4.49±0.97 | 2.31±0.70 | 24.82±0.59 | 10.05±0.23 | 6.91±0.01 | 0.83±0.07 | 13.93±0.82 | 36.67±1.25 |
| 4097-2 | 3.31±0.19 | 1.97±0.30 | 37.25±1.27 | 11.60±0.45 | 5.94±0.29 | 0.99±0.10 | 18.73±0.16 | 20.20±0.59 |
| 4111-2 | 3.46±0.11 | 1.60±0.06 | 23.64±1.70 | 12.73±0.19 | 7.70±0.05 | 0.65±0.03 | 12.47±0.45 | 37.74±2.18 |
| 768 | 5.43±0.58 | 3.23±0.71 | 44.61±7.55 | 14.98±1.19 | 6.39±0.14 | 0.51±0.07 | 12.78±2.79 | 12.09±5.58 |
| 873 | 4.25±0.29 | 2.71±0.34 | 72.58±0.49 | 14.52±0.13 | 4.70±0.23 | 0.41±0.01 | 0.68±0.05 | 0.15±0.05 |
| 891 | 6.07±0.34 | 4.00±0.01 | 64.92±0.58 | 15.59±0.30 | 7.47±0.19 | 0.60±0.06 | 1.15±0.02 | 0.20±0.18 |
| 8339 | 5.49±0.78 | 3.04±0.54 | 66.13±1.01 | 17.10±0.54 | 6.84±0.17 | 0.43±0.03 | 0.77±0.03 | 0.20±0.08 |
| 974-1 | 4.68±0.16 | 2.18±0.34 | 72.51±0.92 | 13.28±0.34 | 5.68±0.30 | 0.48±0.03 | 1.06±0.08 | 0.12±0.06 |
| 974-2 | 5.22±1.19 | 2.7±0.81 | 70.09±1.21 | 13.33±0.57 | 6.77±0.31 | 0.50±0.04 | 1.02±0.07 | 0.38±0.09 |
| 9005 | 6.53±1.75 | 4.02±1.37 | 47.22±3.65 | 14.59±0.56 | 5.95±0.31 | 0.51±0.10 | 12.93±2.92 | 8.26±3.11 |
| 9009 | 4.57±0.73 | 2.53±0.27 | 58.29±7.20 | 15.79±0.60 | 6.29±1.30 | 0.44±0.02 | 7.61±3.79 | 4.47±2.52 |
| 9007-1 | 4.02±0.43 | 1.82±0.11 | 61.62±2.89 | 17.81±0.17 | 7.64±0.59 | 0.46±0.04 | 4.43±1.82 | 2.20±1.15 |
| Zhongshuang 11 | 4.10±0.05 | 2.25±0.22 | 65.14±0.91 | 18.81±0.13 | 8.13±1.05 | 0.58±0.04 | 0.97±0.03 | 0.02±0.00 |
| Zheshuang 72 | 4.43±0.67 | 2.42±0.68 | 57.47±3.63 | 18.04±0.74 | 7.74±0.09 | 0.48±0.08 | 3.99±2.34 | 5.44±1.93 |
| Zheyou 50 | 5.51±0.78 | 3.83±0.67 | 65.47±1.27 | 17.09±0.27 | 6.83±0.33 | 0.42±0.02 | 0.74±0.06 | 0.11±0.08 |
| Zheyou 51 | 5.29±0.34 | 3.08±0.12 | 64.01±0.27 | 17.73±0.50 | 8.54±0.21 | 0.43±0.06 | 0.74±0.05 | 0.17±0.16 |
| Qinyou 2 | 5.06±1.37 | 3.03±0.85 | 56.81±3.12 | 14.42±0.29 | 7.86±0.43 | 0.55±0.03 | 6.61±1.60 | 5.64±2.33 |
| Zhongyouza 19 | 4.00±0.29 | 2.65±0.39 | 66.01±0.54 | 17.54±0.06 | 8.33±0.15 | 0.51±0.01 | 0.90±0.03 | 0.07±0.03 |
| Jiayou 1 | 4.69±0.33 | 2.91±0.39 | 58.98±5.96 | 16.60±0.31 | 8.20±0.35 | 0.54±0.05 | 4.17±2.77 | 3.91±3.54 |
| Shanyouza 1 | 4.41±0.41 | 3.35±0.22 | 68.28±2.00 | 15.80±1.09 | 6.73±0.77 | 0.47±0.04 | 0.90±0.03 | 0.06±0.03 |
| Youyan 818 | 4.41±0.41 | 2.97±0.33 | 67.28±1.95 | 15.58±1.24 | 7.84±0.66 | 0.62±0.04 | 1.11±0.03 | 0.19±0.16 |

**Supplementary Table S3** Information on 44 genotypes in this study

| Number | Name | Origin | Haplotype |
| --- | --- | --- | --- |
| 1 | 634 | Prof. Weijun Zhou | A |
| 2 | 941 | Prof. Weijun Zhou | A |
| 3 | 20CP75 | Dr. Qi Peng | A |
| 6 | 20CP86 | Dr. Qi Peng | A |
| 4 | 20CP102 | Dr. Qi Peng | A |
| 5 | 20CP103 | Dr. Qi Peng | A |
| 7 | 21L10 | Inbred line | A |
| 8 | 4095-2 | Prof. Weijun Zhou | A/T |
| 9 | 4099 | Prof. Weijun Zhou | A/T |
| 10 | 612 | Prof. Weijun Zhou | A/T |
| 11 | Zhongyou 821 | Commercial varieties | A/T |
| 12 | Zhongyou 828 | Commercial varieties | A/T |
| 13 | Fengyou 737 | Commercial varieties | A/T |
| 14 | Zhengyou 1 | Commercial varieties | A/T |
| 15 | Zhengyouza 1 | Commercial varieties | A/T |
| 16 | 4017 | Prof. Weijun Zhou | T |
| 17 | 4023 | Prof. Weijun Zhou | T |
| 18 | 4054 | Prof. Weijun Zhou | T |
| 19 | 4064 | Prof. Weijun Zhou | T |
| 20 | 4066 | Prof. Weijun Zhou | T |
| 21 | 4067 | Prof. Weijun Zhou | T |
| 22 | 4068 | Prof. Weijun Zhou | T |
| 23 | 4088 | Prof. Weijun Zhou | T |
| 24 | 4089 | Prof. Weijun Zhou | T |
| 25 | 4097-2 | Prof. Weijun Zhou | T |
| 26 | 4111-2 | Prof. Weijun Zhou | T |
| 27 | 768 | Prof. Weijun Zhou | T |
| 28 | 873 | Prof. Weijun Zhou | T |
| 29 | 891 | Prof. Weijun Zhou | T |
| 30 | 8339 | Prof. Weijun Zhou | T |
| 31 | 974-1 | Prof. Weijun Zhou | T |
| 32 | 974-2 | Prof. Weijun Zhou | T |
| 33 | 9005 | Prof. Weijun Zhou | T |
| 34 | 9009 | Prof. Weijun Zhou | T |
| 35 | 9007-1 | Prof. Weijun Zhou | T |
| 36 | Zhongshuang 11 | Commercial varieties | T |
| 37 | Zheshuang 72 | Commercial varieties | T |
| 38 | Zheyou 50 | Commercial varieties | T |
| 39 | Zheyou 51 | Commercial varieties | T |
| 40 | Qinyou 2 | Commercial varieties | T |
| 41 | Zhongyouza 19 | Commercial varieties | T |
| 42 | Jiayou 1 | Commercial varieties | T |
| 43 | Shanyouza 1 | Commercial varieties | T |
| 44 | Youyan 818 | Commercial varieties | T |

**Supplementary Table S4** Information on primer sequences (the restriction enzyme sites are underlined)

| Gene | Primer sequence 5’-3’ | Length (bp) | Application |
| --- | --- | --- | --- |
| *BnaGPAT9-A1* | FP: ATGAGCAGCA CGGCAGGAAA | 1131 | Gene clone for *BnaGPAT9-A1* |
|  | RP: TCACTTGTCT TCCAATCTAG CCAGG |  |  |
|  | FP: AGGAGAATTCAACATGAGCAGCACGG |  | Construction of yeast genetic complementation vector |
|  | RP: CGTCCTCGAGTCACTTGTCTTCCAATC |  |  |
|  | FP: AGACAAACCCAAAACAAGCG | 120 | qRT‒PCR for detecting tissue-specific expression of *BnaGPAT9-A1* |
|  | RP: GGTGTCTGGTTTTGTACCTCAAGA |  |  |
| *BnaGPAT9-A10* | FP: ACTACCACAACCAATTCACCC | 1116 | Gene clone for *BnaGPAT9-A10* |
|  | RP: CGTTTACGTTCACTTCTCTTCC |  |  |
|  | FP: GAAATCTAGAAACATGAGCAGTACGGCG |  | Construction of yeast genetic complementation vector |
|  | RP: CATCGGTACCTCACTTCTCTTCCAATCTAG |  |  |
| *BnaGPAT9-C1* | FP: CGATCGGAAGCGAGAGAGAGAGAG | 1131 | Gene clone for *BnaGPAT9-C1* |
|  | RP: GTCTTGTCTAGCAGATTAATGAAACACCAC |  |  |
| *BnaGPAT9-C1^1124T^* | FP: GACCTCTAGAAACATGAGCAGTAC |  | Construction of yeast genetic complementation vector |
|  | RP: CATCGGTACCTCACTTGACTTCCAATCT |  |  |
|  | FP: ATCTCCGGCGATCGGAAGCGAGC | 278 | qRT‒PCR for detecting tissue-specific expression of *BnaGPAT9-C1* |
|  | RP: CAGTTCCAAGGCTCTGGAGG |  |  |
| *BnaGPAT9-C1^1124A^* | FP: GACCTCTAGAAACATGAGCAGTAC |  | Construction of yeast genetic complementation vector |
|  | RP: TAGCCTCGAGTCACTTGTCCTCCAATCT |  |  |
| *BnaGPAT9-C9* | FP: ATGAGCAGTA CGGCGGGGAA | 1116 | Gene clone for *BnaGPAT9-C9* |
|  | RP: TCACTTCTCT TCCAATCTAG CAAGGAGG |  |  |
|  | FP: GAAAGGTACCAACATGAGCAGTACGGC |  | Construction of yeast genetic complementation vector |
|  | RP: GTCCCTCGAGTCACTTCTCTTCCAATC |  |  |
| *BnaGPAT9-C1^1124T^* | FP: GCTCGAATTCCTAACAAGACTTGG | 119 | PARMS for haplotype identification |
|  | RP: GAAGGTCGGAGTCAACGGATTGAAATGATCTTTTGCATTCACTTGA |  |  |
| *BnaGPAT9-C1^1124A^* | FP: GCTCGAATTCCTAACAAGACTTGG | 119 | PARMS for haplotype identification |
|  | RP: GAAGGTGACCAAGTTCATGCTTGAAATGATCTTTGCATTCACTTGT |  |  |
| *Napin-BnaGPAT9-C1^1124A^* | FP: GCATGAGCTCATGAGCAGCAC | 1131 | Construction of p1300-*Napin*-*Nos* vector |
|  | RP: CCGACTGCAGTCACTTGTCTTCCAA |  |  |
| *Napin-BnaGPAT9-C1^1124A^* | FP: CCGAGCTTGAATTGGATCACCC | 117 | Determination of expression level of *BnaGPAT9-C1^1124A^* in transformants |
|  | RP: AGTGAGCGTTGGAGAAATGTCTAGC |  |  |
| *Napin-BnaGPAT9-C1^1124A^* | FP: GTGATCGCCATGCAAATCTCC | 1027 | Transformants identification |
|  | RP: GCCCACGATGTCATGAGTTGC |  |  |
| *BnaUBC9* | FP: GCATCTGCCTCGACATCTTGA | 68 | Reference gene in qRT‒PCR |
|  | RP: CGATAGCAGCACCTTGGAGATA |  |  |


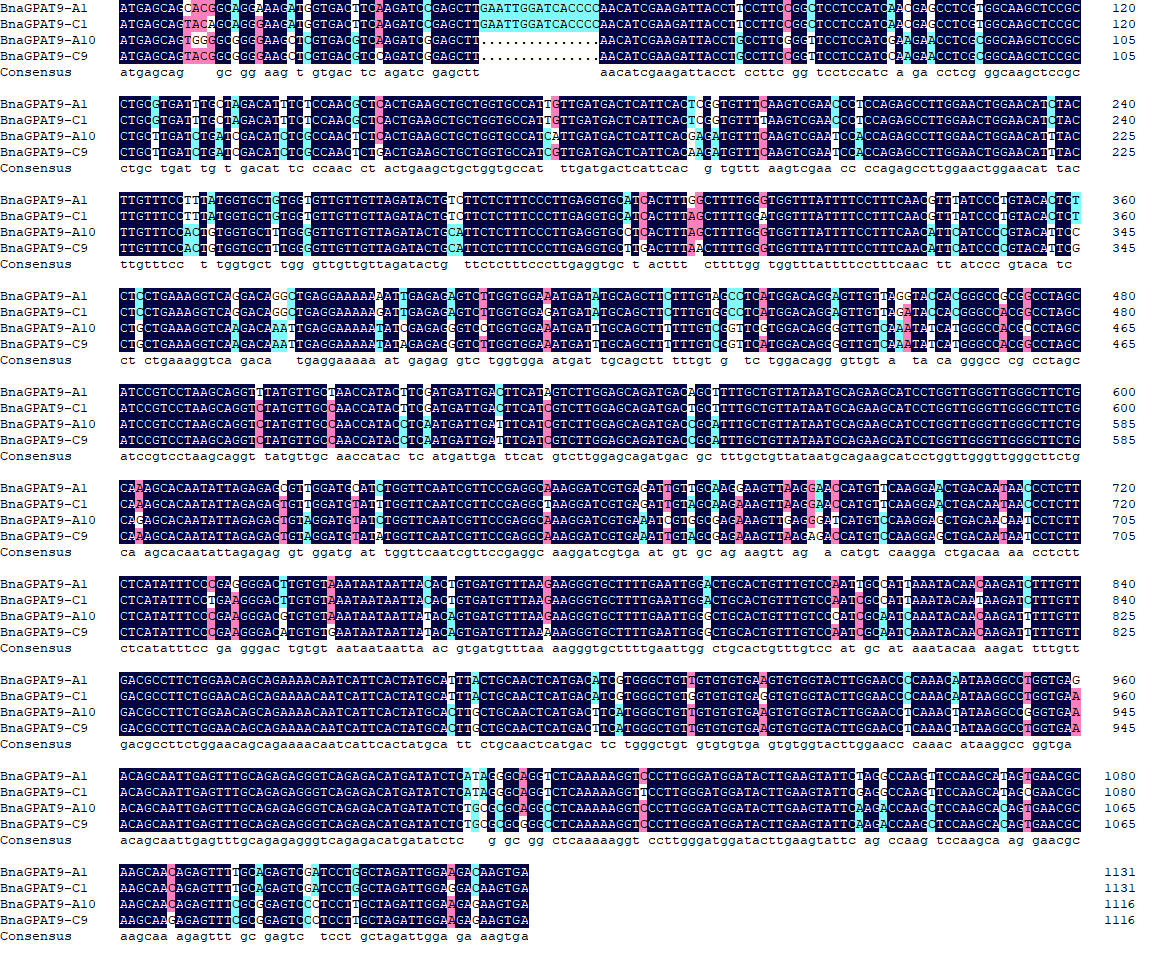


**Supplementary Figure S1** Full-length cDNA sequence alignment of the four homologous *GPAT9* genes in *Brassica napus*. Identical and similar nucleotides are shown in the same colour.


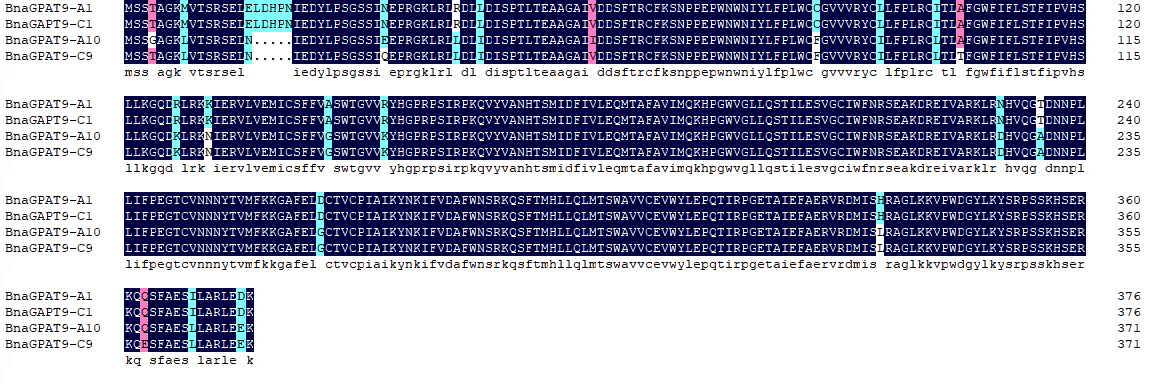


**Supplementary Figure S2** Deduced amino acid sequence alignment of the four homologous GPAT9 proteins in *Brassica napus*. Identical and similar amino acid residues are shown in the same colour.


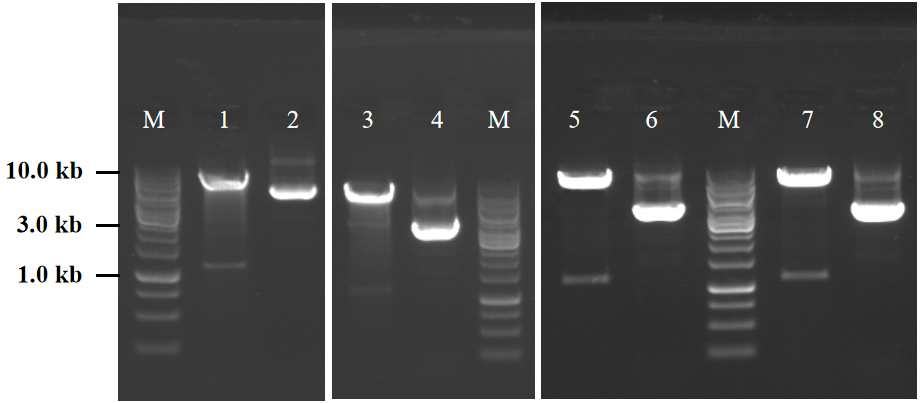


**Supplementary Figure S3** Identification of the recombinant pYES2-Kan-yADH1 V2 vector plus *BnaGPAT9s* by double enzyme digestion. Lanes 1, 3, 5, and 7: digestion of recombinant vector plus *BnaGPAT9-A1*, *C1*, *A10*, and *C9*, respectively; lanes 2, 4: 6, and 8: no digestion of recombinant vector plus *BnaGPAT9-A1*, *C1*, *A10*, and *C9*, respectively; M: 1-kb DNA ladder.


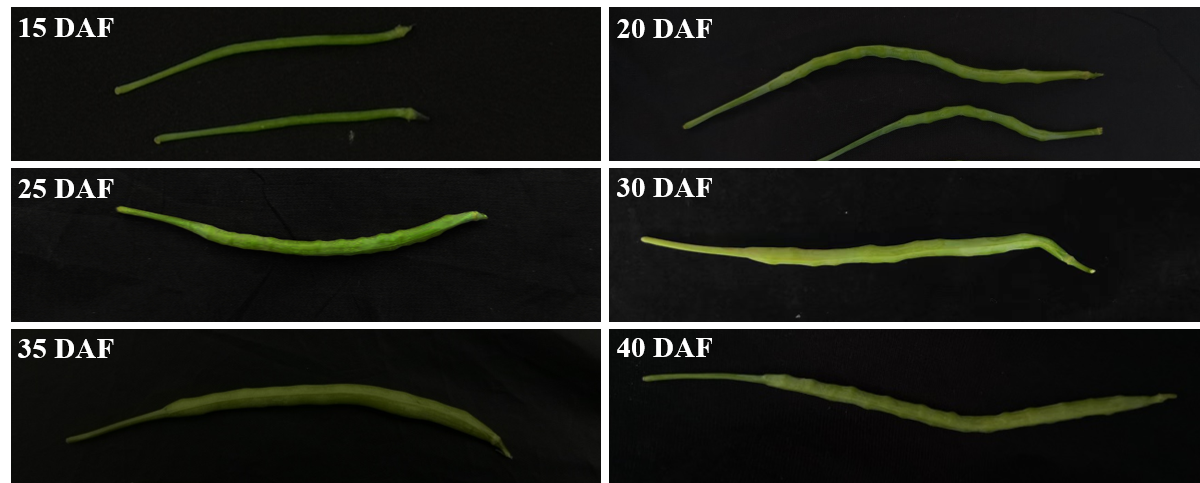


**Supplementary Figure S4** Phenotype of ‘Zhongshuang 11’ developing seeds at 15, 20, 25, 30, 35, and 40 days after flowering (DAF).

**Supplementary Figure S5** Distribution of erucic acid in 44 commercial varieties/advanced lines of three haplotypes.


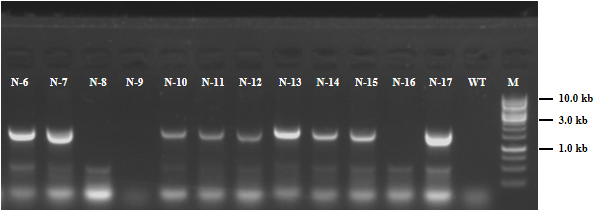


**Supplementary Figure S6** PCR identification of p*Napin*-*BnaGPAT9-C1^1124A^*-*Nos*-independent T_0_ transgenic lines. The target fragment of specific PCR was 1027 bp. N-6, 7, 10, 11, 12, 13, 14, 15, 17: positive plants; N-8, 9, 16: negative plants; WT: ‘Zhongshuang 11’.
